# Efficacy of electroacupuncture on insomnia disorder in older adults: study protocol for a multicentre randomised controlled trial

**DOI:** 10.3389/fneur.2025.1661689

**Published:** 2025-09-02

**Authors:** Jinfeng Mao, Huirong Liu, Zhaoqin Wang, Ming Zhang, Shifen Xu, Jianhua Chen, Wujie Ye, Yuechang Yang, Jing Li, Jianping Li, Yingna Tao, Luyi Wu, Huangan Wu

**Affiliations:** ^1^Yueyang Hospital of Integrated Traditional Chinese and Western Medicine, Shanghai University of Traditional Chinese Medicine, Shanghai, China; ^2^Shanghai Research Institute of Acupuncture and Meridian, Shanghai, China; ^3^Shanghai Chest Hospital, Shanghai Jiao Tong University School of Medicine, Shanghai, China; ^4^Shanghai Municipal Hospital of Traditional Chinese Medicine, Shanghai University of Traditional Chinese Medicine, Shanghai, China; ^5^Shanghai Mental Health Center, Shanghai Jiao Tong University School of Medicine, Shanghai, China; ^6^Jiangsu Province Hospital of Chinese Medicine, Affiliated Hospital of Nanjing University of Chinese Medicine, Nanjing, China; ^7^Shanghai Xuhui District Central Hospital, Shanghai, China; ^8^Shanghai Fourth People’s Hospital, Tongji University, Shanghai, China; ^9^Shanghai University of Traditional Chinese Medicine, Shanghai, China

**Keywords:** acupuncture, insomnia, randomized controlled trial, clinical protocols, older adult

## Abstract

**Introduction:**

Insomnia disorder is highly prevalent among older adults and is associated with significant adverse effects on both physical and psychological health. The use of hypnotic medications in this population is often accompanied by undesirable risks, including drug tolerance, dependence, and impaired daytime functioning. While electroacupuncture has been increasingly employed as a therapeutic modality for insomnia, robust clinical evidence supporting its efficacy and safety remains limited. This multicentre randomised controlled trial is designed to evaluate the therapeutic effectiveness and safety profile of electroacupuncture in the treatment of insomnia disorder in older adults.

**Methods and analysis:**

This multi-centre, single-blind, randomised controlled trial will enrol 214 older adults with diagnosed insomnia disorder from five hospitals. Participants will be randomly assigned in a 1:1 ratio to receive either electroacupuncture or sham electroacupuncture. The electroacupuncture group will undergo treatment three times per week for 8 weeks. The sham electroacupuncture group will receive a non-penetrative placebo needle intervention at sham acupoints, with no electrical stimulation delivered despite connection to the electroacupuncture apparatus. The primary outcome will be the change in Pittsburgh Sleep Quality Index score. Secondary outcomes will include the Insomnia Severity Index, actigraphy-derived sleep parameters, sleep diaries, the Hospital Anxiety and Depression Scale, and the Mini-Mental State Examination. All outcomes will be assessed at baseline (week 0), during intervention (weeks 4 and 8), and during follow-up (weeks 12 and 20).

**Discussion:**

If the intervention demonstrates efficacy, this study will provide important clinical evidence supporting electroacupuncture as a safe and effective non-pharmacological treatment for insomnia in older adults. The findings may influence clinical practice by expanding therapeutic options and improving sleep quality in this vulnerable population.

**Clinical trial registration:**

http://www.chictr.org.cn, identifier ChiCTR2400081782.

## 1 Introduction

Insomnia disorder is clinically defined by persistent disruptions in sleep parameters—such as prolonged sleep latency, frequent nocturnal awakenings, reduced total sleep time, and diminished sleep efficiency—accompanied by significant daytime impairments, including excessive sleepiness, fatigue, somatic complaints, affective instability, and deficits in cognitive function and social engagement (1, 2). Epidemiological studies indicate that approximately 50% of older adults report insomnia symptoms (3, 4), with a meta-analysis involving 18,270 participants estimating a 19.6% prevalence rate of diagnosable insomnia disorder in this population (5). In China, the prevalence is even higher among elderly primary care patients, with one study reporting a rate of 28.9% (6). The vulnerability of older adults to insomnia is particularly pronounced during major public health crises, such as the COVID-19 pandemic (7, 8). Importantly, this elevated prevalence does not suggest that insomnia is a normal consequence of ageing (3). Rather, insomnia in older adults causes substantial physical and psychological distress in older adults and significantly increases the risk of comorbid conditions, including depression, cognitive impairment, and dementia (9–11). Pathophysiologically, insomnia may exacerbate cardiovascular and systemic disorders through dysregulation of neuroendocrine function, metabolic balance, and immune surveillance (12, 13). Given the ongoing global demographic shift toward an aging population (14), the growing burden of insomnia disorder among older adults represents a critical public health issue, adversely affecting quality of life and imposing significant socioeconomic costs (15).

The primary goals in managing insomnia disorder are to enhance sleep quality and/or duration and to mitigate associated daytime dysfunction. Clinical guidelines consistently recommend cognitive behavioural therapy for insomnia (CBT-I) as the first-line intervention (16–18). In older adults, moderate-quality evidence supports the efficacy of CBT-I in improving scores on the Insomnia Severity Index (ISI) and Pittsburgh Sleep Quality Index (PSQI), with low- to moderate-quality evidence indicating improvements in sleep latency, the number of nocturnal awakenings, and sleep efficiency (16). Additionally, CBT-I demonstrates superior long-term efficacy compared to benzodiazepines (BZDs) and non-benzodiazepines (NBZDs) (19). However, the implementation of CBT-I in China remains limited due to several barriers, including a shortage of trained providers, cultural differences between Eastern and Western treatment paradigms, high costs, and a relatively slow onset of therapeutic effects (20, 21). As a result, hypnotic medications—including BZDs, NBZDs, antidepressants, and antipsychotics—continue to be the mainstay of treatment for insomnia disorder in older adults. These agents, however, carry significant risks in this population, such as tolerance, dependence, and adverse events. Notably, 80% of insomnia patients aged 65 years and older report at least one residual side effect from hypnotic use (22). BZDs disrupt REM sleep architecture (23), while both BZDs and NBZDs are associated with increased risks of memory impairment, confusion, falls, and fractures. Long-term use raises the potential for addiction and abuse; consequently, BZDs should be avoided, and NBZDs should not be used for extended durations in older adults (24–26). Antidepressants used in non-depressed elderly patients may further disrupt sleep architecture—suppressing REM and reducing N3 sleep—while also causing anticholinergic and sedative effects and increasing fall risk due to orthostatic hypotension (25). As research into the physiological functions of sleep progresses, questions have emerged regarding the equivalency of pharmacologically induced sleep to natural sleep. For example, evidence suggests that zolpidem disrupts norepinephrine (NE) fluctuations and arterial oscillations, impairing glymphatic flow—a critical mechanism for waste clearance in the brain during sleep—thus diminishing sleep’s restorative function (27).

Acupuncture, a widely utilised non-pharmacological intervention, has been increasingly applied in the clinical management of insomnia disorder, with some underlying mechanisms now partially elucidated (28). In the framework of traditional Chinese medicine (TCM), acupuncture is believed to treat insomnia by restoring the balance of Yin and Yang, calming the mind and spirit (Anshen), and regulating the flow of Qi and blood (29). Our previous clinical studies have demonstrated the efficacy of electroacupuncture (EA) in treating depression-related insomnia (30) and perimenopausal insomnia (31), as well as manual acupuncture for primary insomnia (32). Although preliminary evidence suggests acupuncture may benefit older adults with insomnia, most existing studies are limited by single-centre designs and small sample sizes (33, 34). The specific efficacy of EA for insomnia in older adults remains insufficiently validated and warrants further high-quality, large-scale investigation.

We propose a multi-centre, single-blind, parallel-group, randomised controlled clinical trial to be conducted across five hospitals in Shanghai, China. The study will adopt a standardised EA intervention protocol developed in consensus by experienced acupuncturists. A sham electroacupuncture (SEA) apparatus with validated manufacturing standards and demonstrated usability in prior clinical trials will serve as the placebo control (35–37), with sham acupoints selected in accordance with established practices (38–40). To ensure methodological rigour and scientific validity, the trial will incorporate robust randomisation procedures, EA administration by acupuncturists with over three years of clinical experience and standardised training, and uniform data collection and management protocols overseen by dedicated data monitors. The findings of this study will provide critical evidence regarding the efficacy and safety of EA for treating insomnia disorder in older adults.

## 2 Methods

### 2.1 Trial objective

This trial aims to rigorously evaluate the efficacy and safety of EA in the treatment of insomnia disorder among older adults.

### 2.2 Overall design

**Figure 1:**
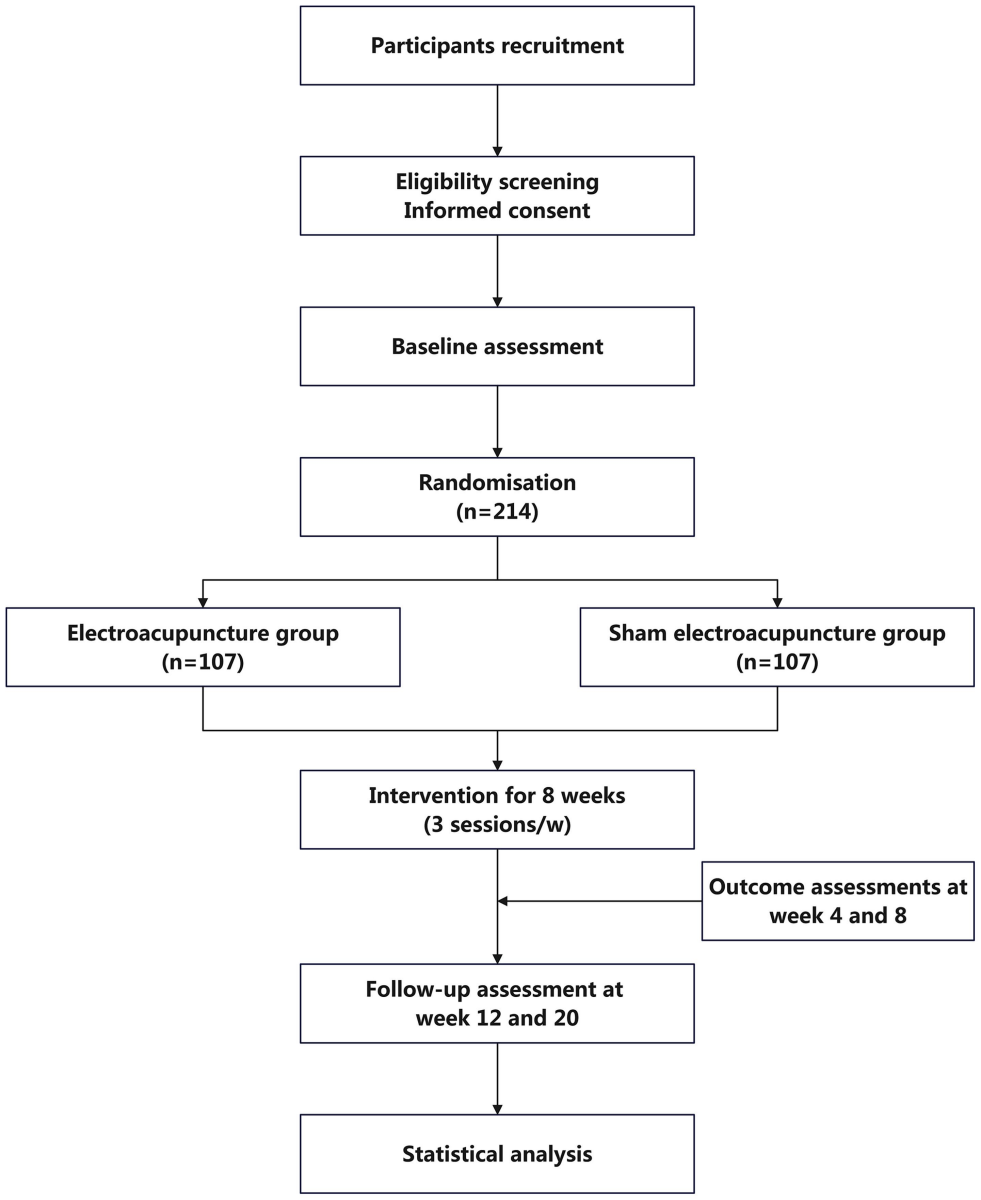
Trial flow chart.

The study is designed as a multi-centre, single-blind, parallel-group, randomised controlled trial. It will be conducted in five hospitals in Shanghai: Yueyang Hospital of Integrated Traditional Chinese and Western Medicine Affiliated to Shanghai University of Traditional Chinese Medicine, Shanghai Municipal Hospital of Traditional Chinese Medicine, Shanghai Mental Health Center, Shanghai Xuhui District Central Hospital, and Shanghai Fourth People’s Hospital. A total of 214 eligible older adults with diagnosed insomnia disorder who provide written informed consent will be randomly assigned in a 1:1 ratio to either the EA group or the SEA control group. Following a one-week baseline assessment, participants will undergo a 20-week observation period, comprising an 8-week intervention phase and a 12-week follow-up phase. EA and SEA interventions will be administered three times per week (on alternate days) for eight weeks. Outcome assessments will be conducted at baseline, at weeks 4 and 8 of intervention, and at weeks 4 and 12 post-intervention. Measures will include the Pittsburgh Sleep Quality Index (PSQI), Insomnia Severity Index (ISI), actigraphy-derived sleep parameters, sleep diaries, Hospital Anxiety and Depression Scale (HADS), and Mini-Mental State Examination (MMSE), among others. Detailed procedures for recruitment, intervention delivery, assessment schedules, and study flow are outlined in Table 1 and Figure 1. The protocol design adheres to the SPIRIT 2013 (Standard Protocol Items: Recommendations for Interventional Trials) guidelines (41).

**Table 1:** Trial process chart.

Time point (week)	Baseline	Intervention			Follow-up	
	0	1	4	8	12	20
Enrollment						
Eligibility screening	×					
Informed consent	×					
Randomization	×					
Interventions						
EA group		24 sessions				
SEA group		24 sessions				
Outcome measurement						
PSQI	×		×	×	×	×
ISI	×		×	×	×	×
Actigraphy	×		×	×		
Sleep diary	×	×	×	×		
HADS	×		×	×	×	×
MMSE	×			×		×
Hypnotic dosage	×	×	×	×		
Usage of an emergency drug		×	×	×		
Blinding assessment				×		
Adverse events		×	×	×		

### 2.3 Ethics statement

The study protocol (Version 1.0, October 6, 2023) was approved by the Ethics Committee of the lead centre, Yueyang Hospital of Integrated Traditional Chinese and Western Medicine Affiliated to Shanghai University of Traditional Chinese Medicine (Approval No: 2023–187), on November 1, 2023. Subsequent ethical approvals were obtained from the ethics committees of the four participating sub-centres: Shanghai Municipal Hospital of Traditional Chinese Medicine (2024SHL-KY-08-01), Shanghai Mental Health Center (2023–91), Shanghai Xuhui District Central Hospital (2023–044), and Shanghai Fourth People’s Hospital (2023117–001). The trial was registered with Chinese Clinical Trial Registry (http://www.chictr.org.cn) on March 12, 2024, under the registration number ChiCTR2400081782.

### 2.4 Recruitment

Recruitment will be conducted via outpatient posters and online platforms, including WeChat official accounts of the participating hospitals. Individuals expressing interest will undergo a two-stage screening process: an initial telephone-based eligibility pre-screening by study coordinators, followed by an in-person assessment at designated clinical centres. Eligible participants who meet all inclusion criteria will be asked to provide written informed consent prior to the intervention. Trained research staff will provide a comprehensive explanation of the study, including its objectives, methodology, potential risks and anticipated benefits, in accordance with the principles of the Declaration of Helsinki to ensure participant autonomy and protection. To minimise dropout, detailed face-to-face consultations will be conducted during recruitment to clarify the study’s time commitment (three sessions per week over eight weeks) and logistical considerations. Only individuals who explicitly confirm their capacity and willingness to comply with the study schedule will be enrolled. A dedicated study coordinator will provide ongoing reminders to support participant adherence and engagement throughout the trial.

### 2.5 Eligibility

#### 2.5.1 Inclusion criteria

Participants must meet all of the following criteria:

1. Aged between 60 and 85 years (inclusive), regardless of gender.
2. Meet the diagnostic criteria for insomnia disorder according to *Diagnostic and Statistical Manual of Mental Disorders, Fifth Edition (DSM-V)* (42).
3. Have a PSQI score > 7.
4. Have not taken hypnotic medications within the past 2 weeks, or are on a stable dose for at least 4 weeks.
5. Provide written informed consent and agree to comply with all intervention procedures and assessments.

#### 2.5.2 Exclusion criteria

Participants will be excluded if any of the following conditions are met:

1. Use of antidepressant or antipsychotic medications within 4 weeks.
2. Evidence of dementia or other severe cognitive impairment (assessed via MMSE score).
3. Diagnosed psychiatric conditions such as depression or anxiety disorders.
4. Neurological disorders such as cerebral infarction or Parkinson’s syndrome.
5. Severe systemic illnesses compromising survival, such as malignancies or advanced malnutrition.
6. Nocturia, pain, or other somatic symptoms identified as the sole contributors to insomnia.
7. Other diagnosed sleep disorders (e.g., obstructive sleep apnoea or restless legs syndrome).
8. History of chronic alcohol abuse or drug dependence.
9. Prior acupuncture treatment for insomnia within the past 6 months or known intolerance to acupuncture.
10. Participation in another clinical trial within the past 3 months.

### 2.6 Sample size calculation

The sample size was determined based on the primary outcome: change in PSQI score from baseline to Week 8. A superiority trial design employing a two-sample mean comparison was used to assess whether EA demonstrates clinically meaningful superiority over SEA. Based on prior reports of the minimal clinically important difference (MCID) for PSQI in traditional Chinese medicine trials (1.69–1.80) (43, 44), the superiority margin(*δ*) was set at 1.8. The sample size per group (n) was calculated using the formula (45):


$$n=[{\left(z_{1-\alpha }+z_{1-\beta }\right)}^{2}\sigma^{2}(1+1/K)]/{\left(\in -\delta \right)}^{2}.$$

where: n is the sample size of per group, σ is the estimated standard deviation, ϵ is the difference between treatment and control group, δ is the superiority margin.

Based on a prior single-centre RCT, PSQI scores decreased by 5.67 in the EA group and 1.83 in the SEA group, yielding ϵ = 3.8 (33). Therefore, we set ϵ = 3.8, assumed a standard deviation σ = 4, a one-sided significance level α = 0.025 (z_1 − α_ = 1.960), a type II error *β* = 0.1 (z_1 − β_ = 1.282), and an allocation ratio K = 1 (1:1 allocation between the experimental and control groups).

The calculated sample size per group is n = 85. Accounting for a 20% attrition rate, the final sample size is adjusted to 107 participants per group, resulting in a total enrolment target of 214 older adults with insomnia disorder.

### 2.7 Randomisation and blinding

Participant randomisation will be conducted via a centralised, web-based system. An independent statistician, uninvolved in trial execution, will generate the allocation sequence using SAS 9.4 software, employing stratified block randomisation with two stratification factors: study site and current use of hypnotic medication. Variable block sizes (2, 4, 6, or) will be used. Following confirmation of eligibility, designated randomization personnel at each study site will input the participant’s information into the central system to obtain a unique identification number and treatment allocation. Access to this system will be restricted to randomisation personnel and data monitors; all other study staff, including outcome assessors and statisticians, will remain blinded. Randomisation personnel will only be able to view participants and allocations within their respective centre.

This trial adopts a single-blind design, with participants blinded to group allocation. Due to the nature of EA, blinding of acupuncturists is not feasible. Participants will be informed during enrolment that they have an equal probability of being assigned to either intervention group. To ensure effective blinding, sham acupuncture needles with validated manufacturing standards and prior use in clinical trials will be used for the SEA group (35–37). Participants will undergo treatment in a supine position while wearing eye masks, and all interventions will be conducted in separate rooms to minimise cross-group contamination. Blinding integrity will be assessed at week 8, after the final treatment session, by asking participants to guess their group assignment. Apart from the acupuncturists administering the intervention, all other study personnel, including outcome assessors and statisticians, will remain blinded until the trial’s conclusion, at which point routine unblinding will be performed. All research staff will undergo comprehensive training prior to trial initiation and will strictly adhere to role separation protocols across departments.

### 2.8 Intervention

Both EA and SEA groups will receive standardised sleep hygiene education in accordance with the Chinese Guidelines for the Diagnosis and Treatment of Adult Insomnia (2017) (46), which includes the following recommendations: ① Avoid consumption of stimulants such as coffee, strong tea, or tobacco within 4–6 h before bedtime; ② Refrain from alcohol use before sleep, particularly as a sedative; ③ Engage in daily moderate physical activity, avoiding intense exercise within 3–4 h before bedtime; ④ Avoid overeating or consuming difficult-to-digest foods prior to bedtime; ⑤ Minimise exposure to stimulating activities, including watching television or reading emotionally arousing material, within 1 h before sleep; ⑥ Maintain a quiet, comfortable, and appropriately lit and temperature-controlled bedroom environment; ⑦ Establish and adhere to a consistent sleep–wake schedule.

Participants in both groups will receive 24 intervention sessions (30 min each), administered three times per week on alternate days over 8 weeks. During each session, participants will lie supine, with intervention areas exposed and eye masks worn to block visual stimuli. Licensed TCM practitioners with over three years of clinical experience, all of whom have completed standardised training specific to this trial, will administer either EA or SEA based on group assignment.

Participants already using hypnotic medications at baseline may continue their prescribed regimens but must record all medication usage. Dose escalation is prohibited unless approved by a psychiatrist. Participants not taking hypnotics at baseline will not be allowed to initiate or increase hypnotic use during the study. In the event of severe insomnia symptoms, such as persistent sleeplessness over multiple nights, participants may use emergency medication—zolpidem tartrate tablets (Stilnox, 5 mg; National Medicine Permit H20181237, Sanofi Winthrop Industrie)—and must document its use. Concurrent insomnia-related treatments, including dietary supplements, Chinese herbal medicines, and CBT-I, are not permitted during the trial.

#### 2.8.1 EA group

**Figure 2:**
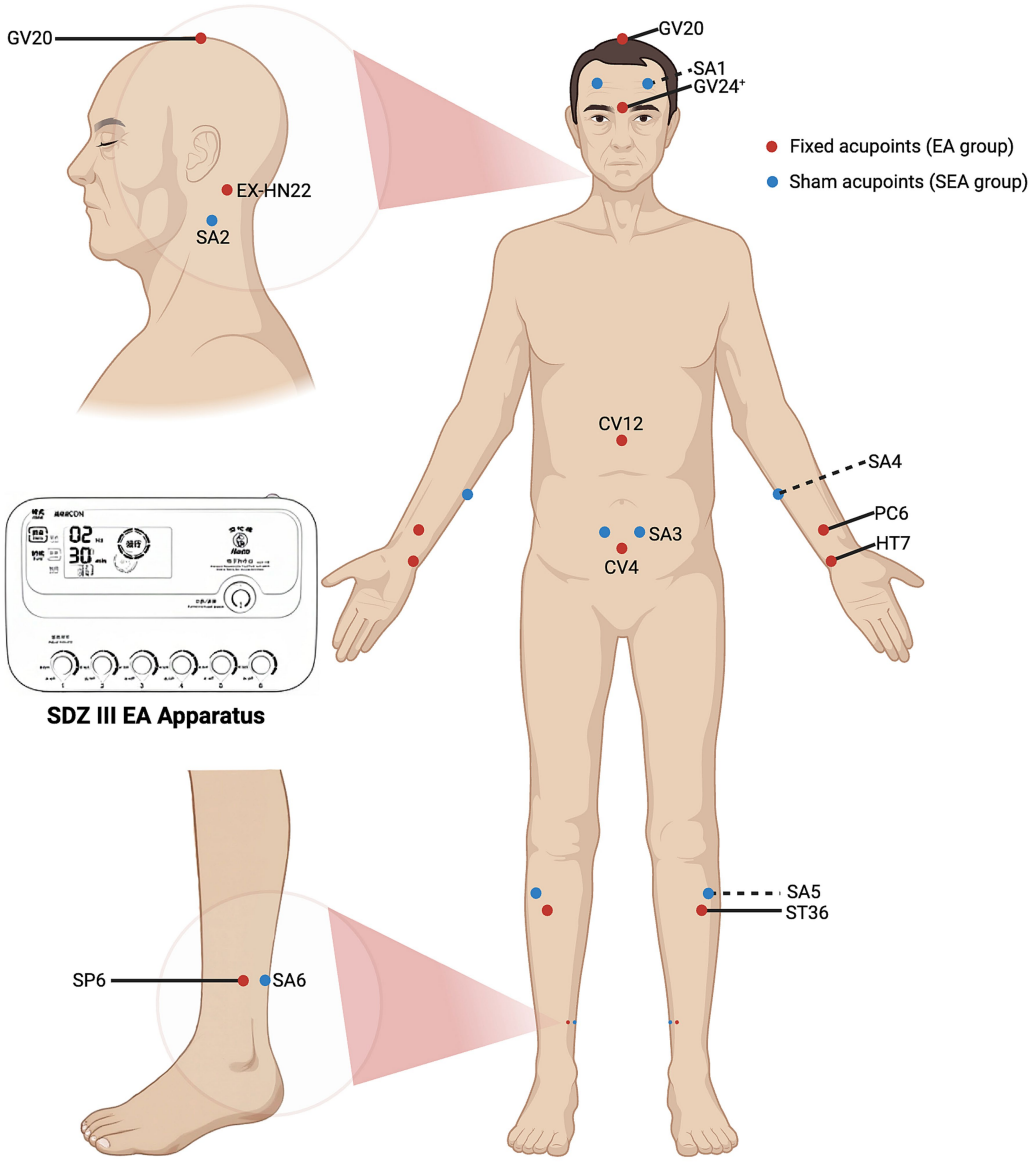
Fixed acupoints for EA group, sham acupoints for SEA group, and the EA apparatus. The red dots indicate the fixed acupoints used in the EA group, including GV20, GV24^+^, EX-HN22, PC6, HT7, CV12, CV4, ST36 and SP6. The blue dots indicate the sham acupoints (SA1-SA6) used in the SEA group. The EA apparatus used is the SDZ-III (Suzhou Medical Appliance Factory Ltd., China).

Participants in the EA group will receive a semi-standardised acupoint protocol. The fixed acupoints used include Baihui (GV20), Yintang (GV24^+^), Anmian (EX-HN22), Shenmen (HT7), Neiguan (PC6), Zhongwan (CV12), Guanyuan (CV4), Zusanli (ST36), and Sanyinjiao (SP6). Additional acupoints will be selected based on comorbid symptoms: Headache/dizziness: Fengchi (GB20), Taichong (LR3); Emotional anxiety symptoms: Taichong (LR3), Shenting (GV24); Depressive symptoms: Danzhong (CV17), Taichong (LR3); Diarrhoea: Tianshu (ST25), Shangjuxu (ST37); Constipation: Tianshu (ST25), Zhigou (TE6). The total number of acupoints will range from 14 to 18 (with bilateral points counted separately). Acupoint locations will follow the Nomenclature and *L*ocation of *Meridian Points* (GB/T 12346–2021) (47) and the Nomenclature and *L*ocation of *Extra Points* in *Common Use* (GB/T 40997–2021) (48). Specific needling techniques are detailed in Table 2. Prior to needling, acupoints will be disinfected and single-use foam pads (Suzhou Medical Appliance Factory Ltd., China)—identical to those used in the SEA group—will be applied. Sterile, single-use Hwato-brand acupuncture needles (0.25 × 40 mm or 0.25 × 25 mm, Suzhou Medical Appliance Factory Ltd., China) will be selected based on the required depth for each acupoint. Needles will be inserted through the foam pads to the appropriate depth, followed by manual manipulation (e.g., lifting, thrusting, rotation) to elicit the *de qi* sensation, characterised by swelling, soreness, numbness, or heaviness. Electrostimulation will then be applied via an SDZ-III EA apparatus (Suzhou Medical Appliance Factory Ltd., China). A diagram of the fixed acupoints and the EA apparatus used is shown in Figure 2. Electrodes will be connected to Baihui and Yintang, and bilaterally to the ipsilateral Zusanli and Sanyinjiao. A continuous wave stimulation at 2 Hz with a current intensity of 1–5 mA (Adjusted for participant comfort and tolerability) will be delivered for 30 min. Following treatment, sterile dry cotton balls will be used to gently compress each acupoint site. Needles will be withdrawn by slowly lifting to the subcutaneous layer and then swiftly removing them, followed by removal of the foam pads.

**Table 2:** Angle and depth of insertion for each acupoint.

Acupoints	Angle and depth of insertion
GV20, GV24	Horizontal insertion, 10-20 mm
GV24^+^, CV17	Horizontal insertion, 5-10 mm
EX-HN22	Perpendicular insertion, 20-30 mm
PC6, LR3, TE6	Perpendicular insertion, 10-25 mm
HT7	Perpendicular insertion, 5-10 mm
CV12	Perpendicular insertion, 20-30 mm
CV4, ST36, SP6, ST25, ST37	Perpendicular insertion, 20-35 mm
GB20	Oblique insertion 20-30 mm toward the tip of the nose

#### 2.8.2 SEA Group

Sham acupoints previously employed in clinical studies will be applied to participants in the SEA group. These points are located anatomically close to true acupoints but lack corresponding therapeutic properties (38–40). The precise locations of the sham acupoints are listed in Table 3, and a diagram of their locations is shown in Figure 2. A SEA device, validated in prior clinical trials (35–37), will be used. It comprises two components: a foam pad affixed to the skin at the sham acupoint, and a custom-made 0.3 × 25 mm blunt-tipped placebo needle (Suzhou Medical Appliance Factory Ltd., China). Following standard skin disinfection, acupuncturists will apply the foam pad and insert the blunt-tipped needle through it, allowing contact with the skin without actual penetration. This produces a pricking sensation mimicking real acupuncture. Next, the three output leads of the SDZ-III EA apparatus will be connected bilaterally to SA1, and ipsilaterally to SA5 and SA6. The device will be set to a continuous 2 Hz waveform. Although the device includes six output ports operated by independent switches, only the three ports not connected to the sham acupoints will be activated, ensuring no electrical stimulation is delivered. To simulate active treatment, the device will emit a sound upon completing the 30-min countdown. At the end of the session, sterile dry cotton balls will be used to press the application sites as the blunt-tipped needles are withdrawn, followed by removal of the foam pads. This procedure is designed to maintain participant blinding by creating the illusion of having received genuine EA treatment.

**Table 3:** Locations of the sham acupoints.

SA	Location
SA1	The midpoint of the line between ST8 (touwei) and EX-HN4 (yuyao)
SA2	The midpoint of the line between SJ16 (tianyou) and SI17 (tianrong)
SA3	2 cun below the center of umbilicus, 1 cun lateral to the anterior median line
SA4	Midpoint of the line between the medial epicondyle of the humerus and the ulnar styloid process
SA5	Midpoint of the line between GB34 (yanglingquan) and ST36 (zusanli)
SA6	1 cm posterior to SP6 (sanyinjiao)

*The locations of ST8, EX-HN4, SJ16, SI17, GB34, ST36 and SP6 are referenced according to the GB/T 12346–2021 and GB/T 40997–2021.

* Cun is a traditional Chinese medicine acupuncture measurement unit used to determine acupoint locations; the width of the participant’s own thumb at the interphalangeal joint is approximately 1 cun.

### 2.9 Outcome measurement

#### 2.9.1 Primary outcome

**PSQI** is a validated, widely used tool for assessing sleep disorders (49). It is easy to administer and demonstrates high reliability and validity (50). The PSQI evaluates sleep quality over the previous month in individuals with organic or non-organic sleep disorders. It comprises 23 items, grouped into seven components: subjective sleep quality, sleep latency, sleep duration, habitual sleep efficiency, sleep disturbances, use of sleep medication, and daytime dysfunction. Each component is scored from 0 to 3, yielding a total PSQI score ranging from 0 to 21. A score > 7 indicates poor sleep quality, with higher scores denoting greater impairment (50).

### 2.9.2 Secondary outcomes

#### 2.9.2.1 ISI

This scale is used to assess the severity of insomnia and treatment outcomes over the past two weeks (51, 52). It includes five items comprising seven questions, each rated on a five-point scale (0–4). The total score ranges from 0 to 28, with clinical interpretation as follows: 0–7, no clinically significant insomnia; 8–14, subthreshold insomnia; 15–21, moderate clinical insomnia; 22–28, severe clinical insomnia.

#### 2.9.2.2 Actigraphy-derived sleep data

The ActiGraph wGT3X-BT (ActiGraph LLC, USA) is a wearable device worn on the non-dominant wrist. It incorporates high-precision accelerometers, gyroscopes, and additional sensors to objectively capture limb movements. This enables the collection of sleep-related parameters, supplementing self-reported sleep diaries. Data will be analysed using the sleep module of ActiLife software version 6.13.6 (ActiGraph LLC, USA) to extract metrics such as total sleep time, sleep efficiency, total wake time, and number of awakenings after sleep onset. Numerous studies have shown strong concordance between actigraphy and polysomnography (PSG) data (53–55). For older adults with insomnia, actigraphy provides a more ecologically valid representation of habitual sleep patterns than PSG, which requires environmental modification and complex leads, thereby making it an effective tool for evaluating clinical efficacy and for follow-up assessments.

#### 2.9.2.3 Sleep diary

The Consensus Sleep Diary is a standardised self-report instrument used to record daily sleep parameters, including bedtime, wake-up time, sleep latency, number of awakenings, wake time after sleep onset, use of hypnotics, and subjective sleep quality ratings (56). From these entries, sleep duration and sleep efficiency can be calculated. Sleep diaries offer insights into subjective sleep experiences and can capture behavioural patterns not readily revealed through objective measures. Their simplicity, ease of use, and capacity for repeated sampling enhance the long-term reliability of sleep assessments.

#### 2.9.2.4 HADS

Developed by Zigmond AS and Snaith RP in 1983 (57), is a widely used self-report tool for assessing symptoms of anxiety and depression. It has demonstrated strong psychometric properties in Chinese populations (58–60). The HADS comprises 14 items, with two subscales: HADS-A (anxiety) and HADS-D (depression), each containing seven items. This study will employ the Chinese version revised by Professor Leung of the Chinese University of Hong Kong (61).

#### 2.9.2.5 MMSE

The MMSE is a widely used tool for screening dementia, assessing the severity of cognitive impairment, and monitoring changes over time. It evaluates seven domains: temporal orientation, spatial orientation, immediate recall, attention and calculation, delayed recall, language, and visuospatial skills, comprising a total of 30 items. Each correct response scores one point, whereas incorrect or “do not know” responses receive zero, yielding a total score ranging from 0 to 30 points (62, 63). In the Chinese version of the MMSE, education-adjusted cut-off scores are commonly applied: ≤17 for illiterate individuals, ≤20 for those with primary education, and ≤24 for those with middle school education or above. This study will employ the Chinese revised version developed by Zhang Mingyuan.

#### 2.9.2.6 Hypnotic medication use

Participants will document their use of hypnotic medications in sleep diaries. Upon data collection, the proportion of participants using hypnotics in both groups before and after the intervention, as well as the proportion with increased or decreased dosages, will be calculated.

#### 2.9.2.7 Blinding assessment

Before trial initiation, participants will be informed that they have an equal chance of being assigned to either intervention group. Immediately following the intervention period, outcome assessors will evaluate the success of blinding by asking participants to guess their intervention allocation. Blinding efficacy will be quantified using Bang’s Blinding Index (64).

### 2.10 Adverse events

Throughout the trial, adverse events (AEs) will be monitored and recorded by participants, outcome assessors, and acupuncturists using a modified acupuncture adverse event recording form. Based on the potential association with the EA or SEA intervention, all AEs will be reviewed and classified as intervention-related or non–intervention-related by the acupuncturist in consultation with relevant experts within 24 h of occurrence. Common AEs associated with EA and SEA include severe pain, subcutaneous hematoma, skin reactions, syncope, and other discomforts. Clinically significant abnormalities in vital signs or laboratory findings will also be recorded as AEs. Appropriate clinical management will be provided according to the severity of the event, and detailed records will be maintained. The incidence of AEs will be reported as the ratio of the total number of events to the total number of intervention sessions.

### 2.11 Data collection and management

Data collection will be conducted by assessors at each clinical site who are independent of both intervention delivery and statistical analysis. All scale-based assessments will be performed in separate rooms to ensure privacy and reduce bias. Assessors, who are blinded to the participants’ group allocation, will undergo standardised training and will provide only necessary clarifications on assessment tools, without influencing participant responses. At each assessment time point, both assessors and participants will be limited to the specific scale being administered. Participants will complete their sleep diaries and promptly submit them to the assessors. Actigraphy data will be downloaded and analysed by trained assessors using ActiLife software.

Recognising the challenges of ensuring accurate and complete self-recording of sleep diaries and actigraphy data, a comprehensive compliance strategy will be implemented. This includes in-person training at baseline on the use of sleep diaries and actigraphy devices, with reinforcement sessions at weeks 4 and 8. A robust monitoring protocol will also be in place, whereby assessors will routinely verify data completeness and accuracy, providing immediate feedback and reminders to participants as needed.

All study data will be initially documented in standardised, paper-based Case Report Forms (CRFs) and subsequently entered into a web-based Electronic Data Capture (EDC) system. Double data entry will be employed to minimise errors. Data entry personnel at each site will remain uninvolved in other trial procedures and will have access only to data from their respective centres. An independent Data and Safety Monitoring Board (DSMB) will oversee data integrity and safety. If discrepancies arise, a data supervisor will issue a query form to the responsible researcher for clarification. If required, the statistician may also initiate a query. All responses will be recorded on the query form, which will then be returned to the statistician by the trial inspector.

### 2.12 Statistical analysis

Statistical analyses will be conducted using SAS version 9.4 (SAS Institute, Cary, NC). Descriptive statistics will be reported as mean ± standard deviation (Mean ± SD) for normally distributed continuous variables with homogeneous variance, and as median with interquartile range [Median (P25, P75)] for non-normally distributed or heteroscedastic data. Categorical variables will be presented as frequencies and percentages. All analyses will follow the intention-to-treat (ITT) principle. Missing data due to participant dropout will be handled using the last observation carried forward (LOCF) method.

Baseline characteristics, including demographic variables and hypnotic medication use before and after the intervention, will be analysed as follows: Continuous data with normal distribution and homogeneity of variance will be assessed using the independent two-sample t-test; otherwise, the Mann–Whitney U test will be applied. Categorical variables will be analysed using the chi-squared test. For the primary and secondary outcome measures, repeated measures will be analysed using a linear mixed-effects model. Fixed effects will include intervention group, time, and their interaction (intervention × time), while a random intercept for each participant will be included as a random effect. Results will be reported as point estimates with 95% confidence intervals. All statistical tests will be two-sided, with a *p*-value < 0.05 considered statistically significant.

To assess potential heterogeneity in treatment effects and enhance the generalisability of the findings, exploratory subgroup analyses will be conducted based on key baseline characteristics. In particular, we will examine whether the primary and selected secondary outcomes differ among participants with common age-related comorbidities such as hypertension and diabetes. These analyses will be exploratory in nature and are intended to inform hypothesis generation for future studies.

## 3 Discussion

Age-related alterations in sleep–wake cycles and circadian rest-activity rhythms often present as fragmented nocturnal sleep and excessive daytime sleepiness in older individuals (65). While multiple clinical guidelines recommend non-pharmacological therapies—particularly CBT-I—as the first-line treatment (16, 17), accessibility to CBT-I remains limited in regions such as China (66), and its efficacy in elderly populations requires further substantiation. Consequently, hypnotic medications remain the primary treatment for insomnia disorder in older adults. However, diminishing therapeutic effects and concerns over adverse outcomes associated with long-term use warrant alternative therapeutic strategies. Acupuncture, a widely utilised non-pharmacological intervention, lists insomnia among its principal indications and is a promising avenue of research (67, 68). Nevertheless, definitive evidence on its efficacy and safety in older adults is lacking, necessitating rigorously designed, adequately powered multicentre randomised controlled trials—such as the present study. Insomnia disorder is now widely recognised as a distinct clinical entity that warrants independent diagnosis and treatment, irrespective of comorbid conditions (42). An expanding body of empirical evidence supports the effectiveness of insomnia-specific interventions even in the presence of comorbidities (69). Given the complex health profiles of older adults, many of whom present with chronic illnesses, we have implemented exclusion criteria to eliminate confounding conditions such as other sleep disorders, neuropsychiatric illnesses, cancer, or other life-threatening diseases. However, we have not excluded individuals with stable chronic diseases. This decision is based on two considerations: the high prevalence of chronic conditions in this population, which should be balanced across groups through randomisation; and the need to ensure ecological validity by maintaining a sample representative of the broader population of older adults with insomnia. As this is not a pharmacological trial, participants may either be using hypnotic medications regularly or not at all, provided their insomnia symptoms are chronic and stable, and they agree not to increase their medication use after enrolment. To ensure baseline equivalence, pre-enrolment hypnotic use will serve as a stratification factor. This inclusion criterion is consistent with acupuncture research conventions (30, 70, 71) and permits a meaningful evaluation of acupuncture’s therapeutic effect. Based on expert clinical consensus and our prior research, we have selected the acupoints and EA parameters for this trial. Fixed acupoints, derived from traditional Chinese medicine theory and clinical practice, include GV20, GV24^+^, EX-HN22, HT7, PC6, CV12, CV4, ST36, and SP6. In addition, following standard clinical acupuncture practice (72), acupuncturists will select adjunctive acupoints tailored to individual symptoms commonly observed in older adults with insomnia, potentially enhancing treatment efficacy. Given the typically prolonged duration and increased severity of insomnia in older adults, the intervention phase will last 8 weeks, with sessions conducted thrice weekly. A 12-week post-intervention follow-up will assess sustained effects. Assessments will occur at baseline, weeks 4 and 8 of intervention, and weeks 4 and 12 post-intervention (i.e., study weeks 12 and 20), allowing for the evaluation of both short-term and long-term outcomes. This follow-up structure aligns with previous randomised controlled trials investigating acupuncture for other forms of insomnia (30, 32, 70).

Despite its strengths, the study faces certain limitations and implementation challenges. First, due to the nature of acupuncture, a double-blind design is not feasible. To address this, we will implement Standard Operating Procedures (SOPs) for intervention delivery, standardising needling techniques and controlling practitioner-participant interactions, while ensuring strict separation between intervention, assessment, and data analysis teams. Second, the extended duration and high frequency of EA sessions, combined with requirements for participants to maintain sleep diaries and wear actigraphy devices, may affect adherence and contribute to attrition. Third, the success of the blinding procedure relies on participants’ perceptions, which may vary and influence outcomes. Future research should consider incorporating objective biomarkers or extended follow-up periods to better assess the durability and mechanism of treatment effects.

## References

[1] Morin CM Buysse DJ. Management of Insomnia. N Engl J Med. (2024) 391:247–58. doi: 10.1056/NEJMcp2305655, PMID: 39018534

[2] Perlis ML Posner D Riemann D Bastien CH Teel J Thase M. Insomnia. Lancet. (2022) 400:1047–60. doi: 10.1016/S0140-6736(22)00879-0, PMID: 36115372

[3] Brewster GS Riegel B Gehrman PR. Insomnia in the older adult. Sleep Med Clin. (2022) 17:233–9. doi: 10.1016/j.jsmc.2022.03.004, PMID: 35659076

[4] Ohayon MM. Epidemiology of insomnia: what we know and what we still need to learn. Sleep Med Rev. (2002) 6:97–111. doi: 10.1053/smrv.2002.0186, PMID: 12531146

[5] Endomba FT Tchebegna PY Chiabi E Angong Wouna DL Guillet C Chauvet-Gélinier JC. Epidemiology of insomnia disorder in older persons according to the diagnostic and statistical manual of mental disorders: a systematic review and meta-analysis. Eur Geriatr Med. (2023) 14:1261–72. doi: 10.1007/s41999-023-00862-2, PMID: 37725311

[6] Zhong B-L Li H-J Xu Y-M Jiang X-F. Clinical insomnia among elderly primary care attenders in Wuhan, China: a multicenter cross-sectional epidemiological study. Front Public Health. (2022) 10:1026034. doi: 10.3389/fpubh.2022.1026034, PMID: 36339226 PMC9634545

[7] Xu Y-M Li C Zhu R Zhong B-L. Prevalence and correlates of insomnia symptoms in older Chinese adults during the COVID-19 outbreak: a classification tree analysis. J Geriatr Psychiatry Neurol. (2022) 35:223–8. doi: 10.1177/08919887221078561, PMID: 35245996 PMC8899830

[8] Zhang Q-Q Li L Zhong B-L. Prevalence of insomnia symptoms in older Chinese adults during the COVID-19 pandemic: a Meta-analysis. Front Med. (2021) 8:779914. doi: 10.3389/fmed.2021.779914, PMID: 34869501 PMC8634335

[9] Baglioni C Battagliese G Feige B Spiegelhalder K Nissen C Voderholzer U . Insomnia as a predictor of depression: a meta-analytic evaluation of longitudinal epidemiological studies. J Affect Disord. (2011) 135:10–9. doi: 10.1016/j.jad.2011.01.011, PMID: 21300408

[10] Liang Y Qu L-B Liu H. Non-linear associations between sleep duration and the risks of mild cognitive impairment/dementia and cognitive decline: a dose-response meta-analysis of observational studies. Aging Clin Exp Res. (2019) 31:309–20. doi: 10.1007/s40520-018-1005-y, PMID: 30039452

[11] Cavaillès C Berr C Helmer C Gabelle A Jaussent I Dauvilliers Y. Complaints of daytime sleepiness, insomnia, hypnotic use, and risk of dementia: a prospective cohort study in the elderly. Alzheimer's Res Ther. (2022) 14:12. doi: 10.1186/s13195-021-00952-y, PMID: 35057850 PMC8780361

[12] Li M Zhang X-W Hou W-S Tang Z-Y. Insomnia and risk of cardiovascular disease: a meta-analysis of cohort studies. Int J Cardiol. (2014) 176:1044–7. doi: 10.1016/j.ijcard.2014.07.284, PMID: 25156850

[13] Chang H-C Hsu Y-H Chou M-Y Chu C-S Su C-S Liang C-K . Insomnia in older adult females is highly associated with metabolic syndrome. Eur Geriatr Med. (2022) 13:203–12. doi: 10.1007/s41999-021-00543-y, PMID: 34291420

[14] Nikolich-Žugich J Goldman DP Cohen PR Cortese D Fontana L Kennedy BK . Preparing for an aging world: engaging biogerontologists, geriatricians, and the society. J Gerontol A Biol Sci Med Sci. (2016) 71:435–44. doi: 10.1093/gerona/glv164, PMID: 26419976 PMC5014189

[15] Ensrud KE Kats AM Schousboe JT Langsetmo L Vo TN Blackwell TL . Multidimensional sleep health and subsequent health-care costs and utilization in older women. Sleep. (2020) 43:zsz230. doi: 10.1093/sleep/zsz230, PMID: 31755954 PMC7017952

[16] Qaseem A Kansagara D Forciea MA Cooke M Denberg TD Clinical Guidelines Committee of the American College of Physicians. Management of chronic insomnia disorder in adults: a clinical practice guideline from the American College of Physicians. Ann Intern Med. (2016) 165:125–33. doi: 10.7326/M15-2175 27136449

[17] Riemann D Baglioni C Bassetti C Bjorvatn B Dolenc Groselj L Ellis JG . European guideline for the diagnosis and treatment of insomnia. J Sleep Res. (2017) 26:675–700. doi: 10.1111/jsr.12594, PMID: 28875581

[18] Chinese Society of Sleep Disorders. Chinese guideline for diagnosis and treatment of insomnia (2023). Chin J Neurol. (2024) 57:560–84. doi: 10.3760/cma.j.cn113694-20240406-00209

[19] Mitchell MD Gehrman P Perlis M Umscheid CA. Comparative effectiveness of cognitive behavioral therapy for insomnia: a systematic review. BMC Fam Pract. (2012) 13:40. doi: 10.1186/1471-2296-13-40, PMID: 22631616 PMC3481424

[20] Jia L He M Yao J. Research status of cognitive behavioral therapy for chronic insomnia. Sichuan Mental Health. (2022) 35:87–91. doi: 10.11886/scjsws20211127001

[21] Hu S Chen Y Sun Q Zhang A Lou S Chen Q . Cognitive behavioral therapy for insomnia in China: a knowledge, attitude, and practice study among medical doctors treating patients with insomnia. Behav Sleep Med. (2025) 23:273–82. doi: 10.1080/15402002.2024.2449354 39804151

[22] Fitzgerald T Vietri J. Residual effects of sleep medications are commonly reported and associated with impaired patient-reported outcomes among insomnia patients in the United States. Sleep Disord. (2015) 2015:607148. doi: 10.1155/2015/607148, PMID: 26783470 PMC4689974

[23] Pagel JF Parnes BL. Medications for the treatment of sleep disorders: an overview. Prim Care Companion J Clin Psychiatry. (2001) 3:118–25. doi: 10.4088/pcc.v03n0303, PMID: 15014609 PMC181172

[24] Tannenbaum C. Inappropriate benzodiazepine use in elderly patients and its reduction. J Psychiatry Neurosci. (2015) 40:E27–8. doi: 10.1503/jpn.140355, PMID: 25903036 PMC4409441

[25] The American Geriatrics Society 2015 beers criteria update expert panel. American Geriatrics Society 2015 updated beers criteria for potentially inappropriate medication use in older adults. J Am Geriatr Soc (2015) 63:2227–2246. doi: 10.1111/jgs.13702 26446832

[26] Kamel NS Gammack JK. Insomnia in the elderly: cause, approach, and treatment. Am J Med. (2006) 119:463–9. doi: 10.1016/j.amjmed.2005.10.051, PMID: 16750956

[27] Hauglund NL Andersen M Tokarska K Radovanovic T Kjaerby C Sørensen FL . Norepinephrine-mediated slow vasomotion drives glymphatic clearance during sleep. Cell. (2025) 188:606–622.e17. doi: 10.1016/j.cell.2024.11.027, PMID: 39788123 PMC12340670

[28] Zhao F-Y Spencer SJ Kennedy GA Zheng Z Conduit R Zhang W-J . Acupuncture for primary insomnia: effectiveness, safety, mechanisms and recommendations for clinical practice. Sleep Med Rev. (2023) 74:101892. doi: 10.1016/j.smrv.2023.101892 38232645

[29] Zhang B Wang Q Zhang Y Wang H Kang J Zhu Y . Treatment of insomnia with traditional Chinese medicine presents a promising Prospect. Phytother Res. (2025) 39:2724–56. doi: 10.1002/ptr.8495, PMID: 40251853

[30] Yin X Li W Liang T Lu B Yue H Li S . Effect of Electroacupuncture on insomnia in patients with depression: a randomized clinical trial. JAMA Netw Open. (2022) 5:e2220563. doi: 10.1001/jamanetworkopen.2022.20563, PMID: 35797047 PMC9264041

[31] Li S Wang Z Wu H Yue H Yin P Zhang W . Electroacupuncture versus sham acupuncture for Perimenopausal insomnia: a randomized controlled clinical trial. Nat Sci Sleep. (2020) 12:1201–13. doi: 10.2147/NSS.S282315, PMID: 33376432 PMC7764880

[32] Yin X Gou M Xu J Dong B Yin P Masquelin F . Efficacy and safety of acupuncture treatment on primary insomnia: a randomized controlled trial. Sleep Med. (2017) 37:193–200. doi: 10.1016/j.sleep.2017.02.012, PMID: 28899535

[33] Wang X-Q Qin S Wu W-Z Liu C-Y Shang H-T Wan Q-Y . Effect of electroacupuncture on serum melatonin and dopamine in aged insomnia. Zhongguo Zhen Jiu. (2021) 41:501–4. doi: 10.13703/j.0255-2930.20200404-k0001, PMID: 34002562

[34] Min J Kim B Park H. The effects of auricular acupressure on the sleep of the elderly using polysomnography, actigraphy and blood test: randomized, single-blind, sham control. Complement Ther Clin Pract. (2021) 45:101464. doi: 10.1016/j.ctcp.2021.101464, PMID: 34352596

[35] Qi L-Y Yang J-W Yan S-Y Tu J-F She Y-F Li Y . Acupuncture for the treatment of diarrhea-predominant irritable bowel syndrome: a pilot randomized clinical trial. JAMA Netw Open. (2022) 5:e2248817. doi: 10.1001/jamanetworkopen.2022.48817, PMID: 36580333 PMC9856830

[36] Pei L Wang G Yang S Zhou S Xu T Zhou J . Electroacupuncture reduces duration of postoperative ileus after laparoscopic gastrectomy for gastric Cancer: a multicenter randomized trial. Gastroenterology. (2025) 169:73–84. doi: 10.1053/j.gastro.2025.02.006, PMID: 39978558

[37] Liu B Xu H Ma R Mo Q Yan S Liu Z. Effect of blinding with a new pragmatic placebo needle. Medicine. (2014) 93:e200. doi: 10.1097/MD.0000000000000200, PMID: 25501074 PMC4602803

[38] Yin X Li W Wu H Dong B Ma J Li S . Efficacy of electroacupuncture on treating depression-related insomnia: a randomized controlled trial. Nat Sci Sleep. (2020) 12:497–518. doi: 10.2147/NSS.S253320 32765146 PMC7382580

[39] Yang J-W Wang L-Q Zou X Yan S-Y Wang Y Zhao J-J . Effect of acupuncture for postprandial distress syndrome: a randomized clinical trial. Ann Intern Med. (2020) 172:777–85. doi: 10.7326/M19-2880, PMID: 32422066

[40] Sun Y Liu Y Liu B Zhou K Yue Z Zhang W . Efficacy of acupuncture for chronic prostatitis/chronic pelvic pain syndrome: a randomized trial. Ann Intern Med. (2021) 174:1357–66. doi: 10.7326/M21-1814 34399062

[41] Chan A-W Tetzlaff JM Altman DG Laupacis A Gøtzsche PC Krleža-Jerić K . SPIRIT 2013 statement: defining standard protocol items for clinical trials. Ann Intern Med. (2013) 158:200–7. doi: 10.7326/0003-4819-158-3-201302050-00583, PMID: 23295957 PMC5114123

[42] American Psychiatric Association. Diagnostic and statistical manual of mental disorders, vol. xliv. 5th ed. Arlington, VA: American Psychiatric Association (2013). 947 p.

[43] Lu T Li Y Pan J Wu D. Study on minimal important difference of the Pittsburgh sleep quality index based on clinical trial of traditional Chinese medicine. J Guangzhou Univ Tradit Chin Med. (2013) 30:574–8. doi: 10.13359/j.cnki.gzxbtcm.2013.04.020

[44] Yin X Dong B Liang T Yin P Li X Lin X . Efficacy and safety of electroacupuncture on treating depression-related insomnia: a study protocol for a multicentre randomised controlled trial. BMJ Open. (2019) 9:e021484. doi: 10.1136/bmjopen-2018-021484, PMID: 31005904 PMC6528016

[45] Chow S-C Shao J Wang H Lokhnygina Y. Sample size calculations in clinical research. 3rd ed. New York: Chapman and Hall/CRC (2017). 510 p.

[46] Chinese Society of Neurology. Guideline for the evaluation and treatment of Insomnia in Chinese adults (2017). Chin J Neurol. (2018) 51:324–35. doi: 10.3760/cma.j.issn.1006-7876.2018.05.002

[47] China National Standardization Administration GB/T 12346-2021: Nomenclature and location of meridian points (2021):8–39.

[48] China National Standardization Administration. GB/T 40997-2021: Nomenclature and location of extra points in common use. (2021):2–3.

[49] Buysse DJ Reynolds CF Monk TH Berman SR Kupfer DJ. The Pittsburgh sleep quality index: a new instrument for psychiatric practice and research. Psychiatry Res. (1989) 28:193–213. doi: 10.1016/0165-1781(89)90047-4, PMID: 2748771

[50] Liu X Tang M Hu L Wang A Wu H Zhao G . Reliability and validity of the Pittsburgh sleep quality index. Chin J Psychiatry. (1996) 29:103–7.

[51] Bastien CH Vallières A Morin CM. Validation of the insomnia severity index as an outcome measure for insomnia research. Sleep Med. (2001) 2:297–307. doi: 10.1016/s1389-9457(00)00065-4, PMID: 11438246

[52] Yu DSF. Insomnia severity index: psychometric properties with Chinese community-dwelling older people. J Adv Nurs. (2010) 66:2350–9. doi: 10.1111/j.1365-2648.2010.05394.x, PMID: 20722803

[53] Smith C Galland B Taylor R Meredith-Jones K. ActiGraph GT3X+ and Actical wrist and hip worn accelerometers for sleep and wake indices in young children using an automated algorithm: validation with polysomnography. Front Psych. (2020) 10:958. doi: 10.3389/fpsyt.2019.00958, PMID: 31992999 PMC6970953

[54] Cellini N Buman MP McDevitt EA Ricker AA Mednick SC. Direct comparison of two actigraphy devices with polysomnographically recorded naps in healthy young adults. Chronobiol Int. (2013) 30:691–8. doi: 10.3109/07420528.2013.782312 23721120

[55] Migueles JH Cadenas-Sanchez C Ekelund U Delisle Nyström C Mora-Gonzalez J Löf M . Accelerometer data collection and processing criteria to assess physical activity and other outcomes: a systematic review and practical considerations. Sports Med. (2017) 47:1821–45. doi: 10.1007/s40279-017-0716-0, PMID: 28303543 PMC6231536

[56] Carney CE Buysse DJ Ancoli-Israel S Edinger JD Krystal AD Lichstein KL . The consensus sleep diary: standardizing prospective sleep self-monitoring. Sleep. (2012) 35:287–302. doi: 10.5665/sleep.1642, PMID: 22294820 PMC3250369

[57] Zigmond AS Snaith RP. The hospital anxiety and depression scale. Acta Psychiatr Scand. (1983) 67:361–70. doi: 10.1111/j.1600-0447.1983.tb09716.x, PMID: 6880820

[58] Yang Y Ding R Hu D Zhang F Sheng L. Reliability and validity of a Chinese version of the HADS for screening depression and anxiety in psycho-cardiological outpatients. Compr Psychiatry. (2014) 55:215–20. doi: 10.1016/j.comppsych.2013.08.012, PMID: 24199886

[59] Zheng L Wang Y Li H. Application of hospital anxiety and depression scale in general hospital: an analysis in reliability and validity. Shanghai Arch Psychiatry. (2003) 15:264–6.

[60] Leung CM Wing YK Kwong PK Lo A Shum K. Validation of the Chinese-Cantonese version of the hospital anxiety and depression scale and comparison with the Hamilton rating scale of depression. Acta Psychiatr Scand. (1999) 100:456–61. doi: 10.1111/j.1600-0447.1999.tb10897.x, PMID: 10626925

[61] Leung CM Ho S Kan CS Hung CH Chen CN. Evaluation of the Chinese version of the hospital anxiety and depression scale. A cross-cultural perspective. Int J Psychosom. (1993) 40:29–34. 8070982

[62] Folstein MF Folstein SE McHugh PR. “Mini-mental state”. A practical method for grading the cognitive state of patients for the clinician. J Psychiatr Res. (1975) 12:189–98. doi: 10.1016/0022-3956(75)90026-6, PMID: 1202204

[63] Robert K Mingyuan Z Ouang-Ya-Qu Wang Z Liu WT Yu E . A Chinese version of the mini-mental state examination; impact of illiteracy in a Shanghai dementia survey. J Clin Epidemiol. (1988) 41:971–8. doi: 10.1016/0895-4356(88)90034-0 3193141

[64] Bang H Ni L Davis CE. Assessment of blinding in clinical trials. Control Clin Trials. (2004) 25:143–56. doi: 10.1016/j.cct.2003.10.016 15020033

[65] Singletary KG Naidoo N. Disease and degeneration of aging neural systems that integrate sleep drive and circadian oscillations. Front Neurol. (2011) 2:66. doi: 10.3389/fneur.2011.00066, PMID: 22028699 PMC3199684

[66] Zhao Y Ge F Luo X Li J Zhang J Ju Y . The applicability and effectiveness of the cognitive behavioral therapy for insomnia (smart CBT-I plus) online program in patients with insomnia disorder combined with anxiety and depression: a randomized controlled trial protocol. Front Psych. (2025) 16:1450275. doi: 10.3389/fpsyt.2025.1450275, PMID: 40225844 PMC11986715

[67] Lu L Zhang Y Tang X Ge S Wen H Zeng J . Evidence on acupuncture therapies is underused in clinical practice and health policy. BMJ. (2022) 376:e067475. doi: 10.1136/bmj-2021-067475, PMID: 35217525 PMC8868048

[68] Lu L Zhang Y Ge S Wen H Tang X Zeng JC . Evidence mapping and overview of systematic reviews of the effects of acupuncture therapies. BMJ Open. (2022) 12:e056803. doi: 10.1136/bmjopen-2021-056803, PMID: 35667716 PMC9171228

[69] McCrae CS Curtis AF Williams JM Dautovich ND McNamara JPH Stripling A . Efficacy of brief behavioral treatment for insomnia in older adults: examination of sleep, mood, and cognitive outcomes. Sleep Med. (2018) 51:153–66. doi: 10.1016/j.sleep.2018.05.018, PMID: 30195661 PMC6513321

[70] Garland SN Xie SX DuHamel K Bao T Li Q Barg FK . Acupuncture versus cognitive behavioral therapy for insomnia in cancer survivors: a randomized clinical trial. J Natl Cancer Inst. (2019) 111:1323–31. doi: 10.1093/jnci/djz050, PMID: 31081899 PMC6910189

[71] Wang C Yang W-J Yu X-T Fu C Li J-J Wang J . Acupuncture for insomnia with short sleep duration: protocol for a randomised controlled trial. BMJ Open. (2020) 10:e033731. doi: 10.1136/bmjopen-2019-033731, PMID: 32139486 PMC7059535

[72] Yang J-W Qi L-Y Yan S-Y She Y-F Li Y Chi L-L . Efficacy of acupuncture in irritable bowel syndrome (ACTION): a multi-Centre randomized controlled trial. Gastroenterology. (2025). doi: 10.1053/j.gastro.2025.05.016 40441496

